# Enhancing breast positioning quality through real-time AI feedback

**DOI:** 10.1007/s00330-025-11812-w

**Published:** 2025-07-15

**Authors:** Raphael Sexauer, Friederike Riehle, Karol Borkowski, Carlotta Ruppert, Silke Potthast, Noemi Schmidt

**Affiliations:** 1 Department of Radiology and Nuclear Medicine, Kantonsspital Baselland, Liestal, Switzerland; 2 Department of Radiology and Nuclear Medicine, University Hospital Basel, Basel, Switzerland; 3 b-RayZ, Schlieren, Switzerland; 4 Institute of Radiology, Limmatthal Hospital, Schlieren, Switzerland

**Keywords:** Deep learning, Mammography, Breast neoplasms, Quality improvement, Feedback

## Abstract

**Objectives:**

Enhance mammography quality to increase cancer detection by implementing continuous AI-driven feedback mechanisms, ensuring reliable, consistent, and high-quality screening by the ‘Perfect’, ‘Good’, ‘Moderate’, and ‘Inadequate’ (PGMI) criteria.

**Materials and methods:**

To assess the impact of the AI software ‘b-box^TM^’ on mammography quality, we conducted a comparative analysis of PGMI scores. We evaluated scores 50 days before (A) and after the software’s implementation in 2021 (B), along with assessments made in the first week of August 2022 (C1) and 2023 (C2), comparing them to evaluations conducted by two readers. Except for postsurgical patients, we included all diagnostic and screening mammograms from one tertiary hospital.

**Results:**

A total of 4577 mammograms from 1220 women (mean age: 59, range: 21–94, standard deviation: 11.18) were included. 1728 images were obtained before (A) and 2330 images after the 2021 software implementation (B), along with 269 images in 2022 (C1) and 250 images in 2023 (C2). The results indicated a significant improvement in diagnostic image quality (*p* < 0.01). The percentage of ‘Perfect’ examinations rose from 22.34% to 32.27%, while ‘Inadequate’ images decreased from 13.31% to 5.41% in 2021, continuing the positive trend with 4.46% and 3.20% ‘inadequate’ images in 2022 and 2023, respectively (*p* < 0.01).

**Conclusion:**

Using a reliable software platform to perform AI-driven quality evaluation in real-time has the potential to make lasting improvements in image quality, support radiographers’ professional growth, and elevate institutional quality standards and documentation simultaneously.

**Key Points:**

***Question*** *How can AI-powered quality assessment reduce inadequate mammographic quality, which is known to impact sensitivity and increase the risk of interval cancers*?

***Findings*** *AI implementation decreased ‘inadequate’ mammograms from 13.31% to 3.20% and substantially improved parenchyma visualization, with consistent subgroup trends*.

***Clinical relevance*** *By reducing ‘inadequate’ mammograms and enhancing imaging quality, AI-driven tools improve diagnostic reliability and support better outcomes in breast cancer screening*.

**Graphical Abstract:**

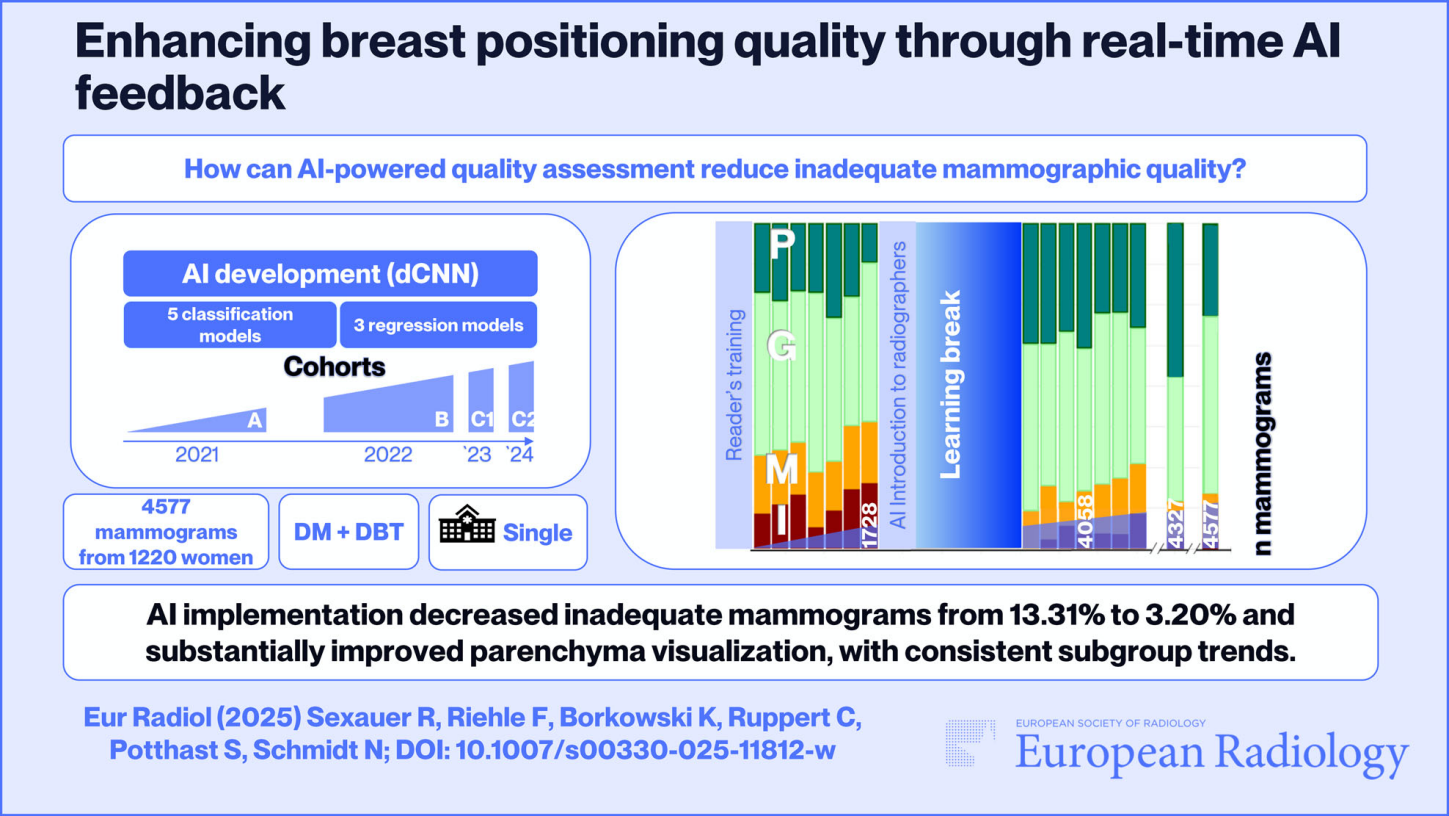

## Introduction

Mammography remains a cornerstone in the early detection and diagnosis of breast cancer, playing an essential role in reducing breast cancer mortality [1]. Mammographic breast cancer screening is the most effective and most widely used method to detect breast cancer in an early stage [2]. The cancer detectability is significantly influenced by the diagnostic image quality, such as inadequate positioning, image artefacts, or insufficient breast compression [3, 4].

To control and evaluate the image quality of mammograms, the perfect-good-moderate-inadequate (PGMI) criteria, established by the United Kingdom Mammography Trainers Group and supported by the Society and College of Radiographers [5], are widely adopted due to their straightforward application and reproducibility [6, 7].

Artificial intelligence (AI) is increasingly used in breast imaging, including diagnostic image quality assessment and workflow optimisation [8–11].

The accurate and consistent visualisation of the entire breast tissue in mammography is essential to the analysis of breast images. In a prior study, we developed an AI system that accurately assessed breast placement and provided immediate feedback on image quality [12].

Therefore, this study evaluates the impact of AI-driven feedback software on the diagnostic quality of mammographic images, aiming to improve reproducibility and consistency in clinical practice.

## Materials and methods

The retrospective longitudinal analysis of prospective cohorts has been approved by the local ethics committee (Project ID 2021-01472 and 2023-01982).

### Patient data

This system expands on our previously published framework [12]. To evaluate the impact of an AI-powered quality assessment system (b-box^TM^, v 1.1, b-rayZ, CE certified Class IIa) introduced on August 1, 2021, four distinct cohorts were defined. Cohort A covered 50 workdays preceding the system’s introduction, while Cohort B spanned 50 workdays following a 30-day learning period (August 1, 2021, to September 10, 2021). These cohorts were subsequently identified as Cohorts A (05/24/2021–07/31/2021) and B (09/11/2021–11/19/2021). Additionally, one year after its introduction (08/01/2022–08/05/2022), and two years after the system’s installation (07/31/2023–08/04/2023), respectively, Cohort C1 and C2 were formed to perform comprehensive longitudinal evaluations of the system’s impact on quality.

During the study period, all consecutive female patients at the University Hospital were considered for inclusion. The device used for the study was the Selenia Dimensions System® (Hologic®). Eligible patients comprised those undergoing screening, screening recall, or diagnostic (symptomatic) imaging, in accordance with routine clinical practice.

Patients undergoing postoperative cancer surveillance, as well as those with pacemakers, surgical clips, or cosmetic breast implants, were excluded from the analysis.

The synthetic 2D images generated from tomosynthesis data were retrieved, anonymised, and stored in a database for subsequent review.

### PGMI criteria and assessment

The European Reference Organisation for Quality Assured Breast Screening and Diagnostic Service provides PGMI criteria, which were used by two readers (Reader 1, N.S.: Board-certified with five years of devoted experience in breast imaging; Reader 2, F.R.: Medical student trained in PGMI) to assess all mammograms. The PGMI criteria were summarised as follows:

- ‘Perfect’: mediolateral oblique (MLO): requires displaying the inframammary fold, the full parenchyma, the nipple in profile, and a clearly defined pectoralis angle. Craniocaudal (CC): Full parenchyma, the nipple in profile, and a sufficiently long posterior nipple line (PNL) or pectoralis muscle are required.
- ‘Good’: a satisfactory level of quality with appropriate positioning and a limited number of artefacts, ensuring breast structures can be viewed clearly.
- ‘Moderate’: adheres to an acceptable range but may require improvement due to minor deficiencies in positioning, artefacts, or clarity deficiencies.
- ‘Inadequate’: does not meet required quality standards; shows important shortcomings in positioning, artefacts, or visualisation; requires further attention or an image re-take.

All mammograms from the database were read in a blinded manner, with PGMI scores, mammography dates, patient data, and the performing radiographer concealed.

### Assurance and training protocols

At our institution, to perform screening mammograms, each radiographer completes a two-day multidisciplinary course, a two-day structured training in mammography acquisition, and a one-week supervised placement at a Swiss partner or European reference screening centre. Prior to AI implementation, routine quality assessment was carried out manually based on PGMI criteria, with continuous, informal feedback from radiologists, a biannual review of at least 20 mammograms per radiographer, and a refresher course every two years to ensure adherence to institutional and national guidelines.

### AI-based software

The ‘b-box^TM^’ AI software utilises deep convolutional neural network (dCNN) models to automatically analyse mammography and 2D synthetic tomosynthesis images (training dataset ratio: DBM:DBT = 1:5). Key aspects of image quality, such as feature localisation and anatomical landmarks, are addressed by eight trained dCNN models. Regression models concentrate on pectoralis and nipple positions, while classification models evaluate parenchyma, the inframammary fold, pectoralis muscle, and nipple depiction.

For implementation, we used the software’s default settings. Specifically, parenchyma in CC was ‘fully depicted’ if the pectoralis muscle was visible or the PNL distance was correct. In MLO, ‘full depiction’ required a correct PNL level, inframammary fold (IMF), and pectoralis muscle angle (≥ 15°). ‘Perfect’ nipple positioning (centred within a 5% tolerance) was achieved only when parenchyma was fully depicted in both views. If parenchyma was fully depicted in only one view, but the nipple was in profile, the depiction was rated as good.

With the system’s real-time feedback feature, radiographers receive PGMI evaluations and key quality criteria (e.g. nipple, inframammary fold) for each mammogram within seconds.

The workflow includes a one-time personal login on a desktop PC, followed by mammogram acquisition with immediate visualisation of quality criteria and PGMI scores. Standard operating procedures (SOPs) were established to guide image acquisition and feedback interpretation. If the software rated an image ‘Inadequate’, the radiographer either retook it or, in case of disagreement, initiated a case discussion with the radiologist.

### Statistical analysis

We used the Mann–Whitney *U*-test to evaluate whether fundamental tendencies differentiate between the two cohorts. A chi-square test of independence was performed to assess the association between the distribution of PGMI scores and time cohorts. To assess interrater agreement, Cohen’s Kappa was employed. According to convention, the following interpretations of Kappa values were made: < 0.00 for poor, 0.00–0.20 for slight, 0.21–0.40 for fair, 0.41–0.60 for moderate, 0.61–0.80 for substantial, and 0.81–1.00 for almost perfect. The application of comparative analysis facilitated the process of quantifying the differences between the cohorts. In addition, we used a mixed-effects model to analyse the impact of cohort and years of experience (YOE) on PGMI scores, accounting for radiographer variability with a random intercept. Akaike information criterion (AIC) was used to compare models with and without YOE, and estimated marginal means (EMM) were calculated to assess adjusted PGMI scores by cohorts. Pairwise comparisons were performed to identify significant differences between cohorts. A significance threshold of *p* < 0.05 was applied. Unless otherwise stated, all results refer to our reference standard (Reader 1). We used R 4.0.5 (R Core Team, R Foundation for Statistical Computing) for statistical analysis.

## Results

**Fig. 1:**
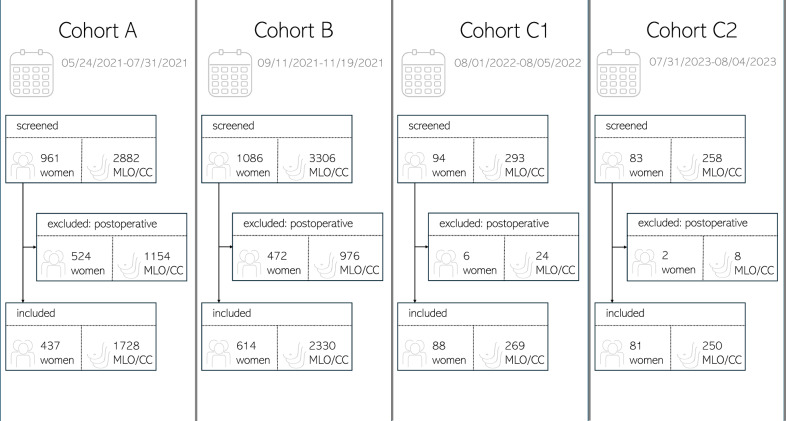
Flowchart summarising the screening, exclusions, and final number of eligible mammograms in Cohorts A, B, C1, and C2

From 961, 1086, 94, and 83 eligible women in Cohorts A, B, C1, and C2, respectively, we excluded 524 women in Cohort A, 472 in Cohort B, 6 in Cohort C1, and 2 in Cohort C2, primarily due to postoperative cancer surveillance or the presence of cosmetic breast implants. A breakdown of examination types across cohorts is available in Supplementary Table S1. Consequently, 1220 women were included (mean age: 59, range: 21–94, standard deviation: 11.18): 437 in Cohort A, 614 in Cohort B, 88 in Cohort C1, and 81 in Cohort C2. These women underwent a total of 4577 mammograms (mean compression: 103.34 N; mean glandular dose: 1.63 mGy; 2D synthetic DBT images: 70.30%), distributed as follows: 1728 in Cohort A, 2330 in Cohort B, 269 in Cohort C1, and 250 in Cohort C2. Detailed information is summarised in Fig. 1.

Post-implementation, standard operating procedures (SOPs) were defined to guide image acquisition and feedback interpretation, including criteria for image repetition and the process for radiographer overrule in case of disagreement.

Over the study period, significant improvements in mammographic positioning quality were observed following the implementation of the AI-powered quality assessment system (see Table 1).

**Table 1:** Quality metrics by PGMI across cohorts

		Cohort A	Cohort B	Cohort C1	Cohort C2
PGMI	Perfect	22.34 (20.39–24.38)	32.27 (30.38–34.22)	47.21 (41.12–53.37)	28.40 (22.90–34.42)
	Good	47.45 (45.08–49.84)	46.78 (44.74–48.83)	37.92 (32.10–44.01)	54.40 (48.01–60.69)
	Moderate	16.90 (15.16–18.75)	15.54 (14.09–17.05)	10.41 (7.03–14.69)	14.00 (9.95–18.93)
	Inadequate	13.31 (11.74–15.50)	5.41 (4.52–6.41)	4.46 (2.33–7.66)	3.20 (1.39–6.21)
Parenchyma	Fully visualized	54.80 (52.42–57.17)	65.92 (63.96–67.85)	72.12 (66.35–77.39)	76.80 (71.07–81.89)
Nipple	In profile	85.88 (84.15–87.49)	88.67 (87.31–89.93)	89.96 (85.73–93.28)	87.20 (82.42–91.08)
Pectoralis angle	Correct	73.50 (70.45–76.33)	63.92 (61.12–66.63)	77.10 (69.19–83.46)	66.67 (57.83–74.47)
	Moderate	23.96 (21.23–26.92)	34.71 (32.03–37.49)	21.37 (15.22–29.16)	30.00 (22.53–38.72)
	Insufficient	2.55 (1.69–3.83)	1.37 (0.85–2.22)	1.53 (0.42–5.40)	3.33 (1.30–8.26)
IMF	Correct	15.16 (12.93–17.71)	17.18 (15.12–19.46)	14.50 (9.49–21.54)	12.50 (7.72–19.60)
	Moderate	39.58 (36.38–42.88)	38.23 (35.48–41.06)	22.14 (15.88–29.98)	15.00 (9.70–22.47)
	Insufficient	45.25 (41.96–48.59)	44.59 (41.75–47.46)	63.36 (54.84–71.12)	72.50 (63.91–79.70)
Pectoralis CC	Correct	25.46 (22.67–28.47)	33.42 (30.77–36.18)	23.53 (17.19–31.32)	36.72 (28.87–45.34)

Percentages of PGMI categories, full parenchyma visualisation, nipple profile alignment, pectoralis in CC, pectoralis angle and IMF in MLO across cohorts before (A), shortly after (B), one (C1) and two (C2) years post-implementation

Values are presented as percentages, with 95% CI in parentheses based on the reference standard, Reader 1

**Fig. 2:**
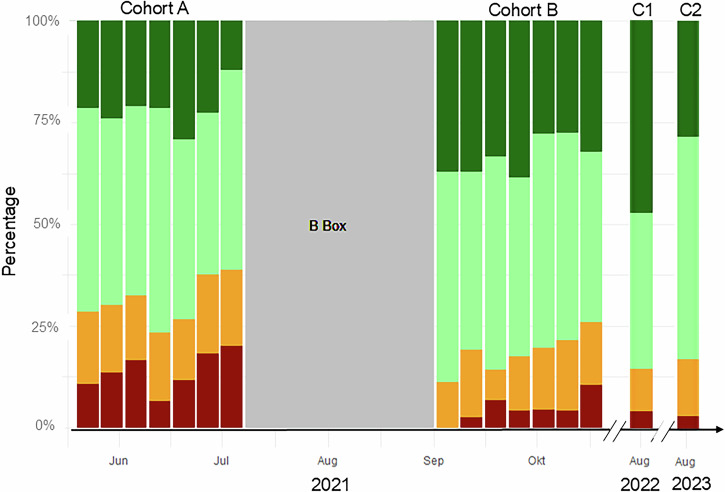
Percentage changes in each PGMI category annotated by Reader 1, from pre-implementation (Cohort A, June–July) to post-implementation (Cohort B, September–November). The sustained improvement is shown over two years (Cohort C1, 2022, and Cohort C2, 2023), highlighting an increase in ‘Perfect’ images and a decrease in ‘Inadequate’ ones

The percentage of ‘Perfect’ mammograms increased notably across all cohorts, while the frequency of ‘Inadequate’ images declined, as depicted in Fig. 2.

### Short-term effect

In 2021 for DBM and DBT a ‘Perfect’ rating increased from 22.34% (*n* = 386, 95% confidence interval (CI): 20.39–24.38%) of all mammograms in Cohort A to 32.27% (*n* = 752, 95% CI: 30.38–34.22%) in Cohort B. Simultaneously, the frequency of ‘Inadequate’ images declined from 13.31% (*n* = 230, 95% CI: 11.74–15.50%) prior to b-box^TM^ implementation to 5.41% (*n* = 126, 95% CI: 4.52–6.41%) in Cohort B. The proportion of ‘Good’ or ‘Perfect’ mammograms rose from 69.79% (*n* = 1206, 95% CI: 67.58–71.91%) to 79.05% (*n* = 1842, 95% CI: 77.36–80.66%). After the AI-based software was implemented, the percentage of ‘Good’ and ‘Moderate’ images exhibited minimal change, maintaining proportions of 47.45% (*n* = 820, 95% CI: 45.08–49.84%) to 46.78% (*n* = 1090, 95% CI: 44.74–48.83%) for ‘Good’ and 16.90% (*n* = 292, 95% CI: 15.16–18.75%) to 15.54% (*n* = 362, 95% CI: 14.09–17.05%) for ‘Moderate’, alongside the annotations of reader 1. Using identical devices, paddles, and mammographic positioning, we observed similar improvements in both digital breast mammography (DBM) and digital breast tomosynthesis (DBT). The percentage of ‘Perfect’ mammograms increased from 22.44% (95% CI: 18.88–26.32%) to 28.75% (95% CI: 25.30–32.38%) in DBM, while in DBT, it rose from 22.30% (95% CI: 19.99–24.74%) to 33.65% (95% CI: 31.39–35.97%). The results of the Mann–Whitney test supported the statistical significance of these findings, resulting in a *p* < 0.01.

In detail, ‘nipple in profile’ slightly improved, from 85.88% (*n* = 1484, Cohort A, 95% CI: 84.15–87.49) to 88.67% (*n* = 2066, Cohort B, 95% CI: 87.31–89.93). In Cohort A, breast parenchyma was fully visualised in 54.80% of mammograms (*n* = 947, 95% CI: 52.42–57.17), compared to 65.92% (*n* = 1536, 95% CI: 63.96–67.85) in Cohort B. The visualisation of the pectoralis muscle increased in the CC view from 25.8% (*n* = 223, 95% CI: 22.82–29.24) pre-implementation to 34.6% (*n* = 388, 31.61–37.82) post-introduction.

**Fig. 3:**
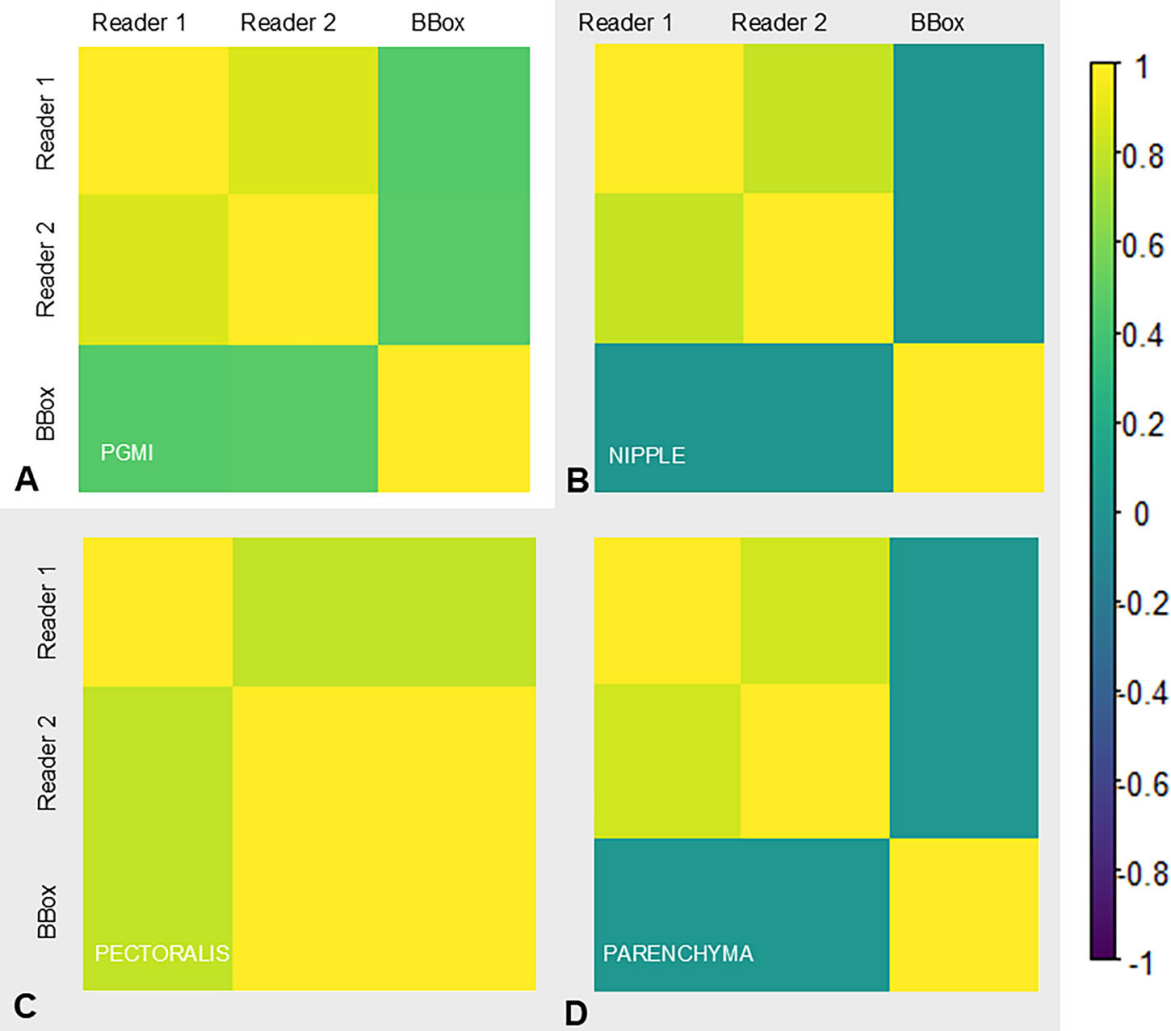
Illustrates the agreement between readers using Kappa coefficients in Cohorts A and B. The Correlation Heatmap depicts the agreement among Reader 1, Reader 2, and b-box^TM^ for (**A**) PGMI, and includes subanalyses for (**B**) nipple in profile, (**C**) correct pectoralis muscle depiction, and (**D**) full parenchyma visualisation

Interrater agreement was fair between b-box^TM^ and Reader 1 (κ = 0.33), while a substantial agreement was observed between Readers 1 and 2 (κ = 0.77). The combined results of the ‘Perfect’ and ‘Good’ categories, as well as the ‘Moderate’ and ‘Inadequate’ categories, yielded fair (k = 0.37) and moderate (k = 0.42) Cohen’s Kappa values for b-box^TM^ vs Reader 1 and b-box^TM^ vs Reader 2. The interrater agreement is summarised for both PGMI and subcategories in Fig. 3.

### Mid-term effect

The distribution of PGMI scores displays a significant difference among the cohorts and time (*p* < 0.01), indicative of a sustained quality improvement. Notably, the percentage of ‘Inadequate’ mammograms decreased from 5.41% (95% CI: 4.52–6.41%) in Cohort B to 4.46% (Cohort C1, 95% CI 2.33–7.66%) and further to 3.20% (Cohort C2, 95% CI: 1.39–6.21%), while the percentage of ‘Moderate’ mammograms remained relatively stable, ranging from 15.54% in 2021 (Cohort B, 95% CI: 14.09–17.05%) to 14.00% in 2023 (Cohort C2, 95% CI: 9.95–18.93%).

The percentage of ‘Perfect’ mammograms in both 2022 and 2023 compared to 2021 (Cohort A), with rates of 47.21% (Cohort C1, 95% CI: 41.12–53.37%) and 28.40% (Cohort C2, 95% CI: 22.90–34.42%) compared to 22.34% (Cohort A, 95% CI: 20.39–24.38%). However, a decline in quality between Cohort C1 and C2 was observed. The cause of this decrease, whether related to the lower mean YOE among radiographers in this cohort (6.75 years vs 5.69 years), remains unclear.

Like PGMI scores, the reported trends were also consistent across subgroup analyses. For instance, the percentage of mammograms with a nipple in profile increased from 85.88% (Cohort A, 95% CI: 84.15–87.49%) to 89.96% (Cohort C1, 95% CI: 85.73–93.28%) and 87.20% (Cohort C2, 95% CI: 82.42–91.08%), while the full visualization of parenchyma increased from 54.80% (Cohort A, 95% CI: 52.42–57.17%) to 72.12% (Cohort C1, 95% CI: 66.35–77.39%) and 76.80% (Cohort C2, 95% CI: 71.07–81.89%). The overall improvement between Cohort A and C2 was +1.32% for nipple visualisation and +22.00% for parenchyma visualisation.

Additionally, interrater agreement demonstrated increasing similarity over time, with a Cohen’s kappa of 0.51 (Cohort C1) and 0.55 (Cohort C2) between b-box^TM^ and Reader 1, and 0.53 (Cohort C1) and 0.58 (Cohort C2) between Reader 1 and 2. However, interrater agreement for Reader 1 vs b-box^TM^ remained fair, with Cohen’s kappa values of 0.26 in Cohort B, 0.32 in Cohort C1, and 0.23 in Cohort C2, comparable to the interrater agreement between Reader 1 and Reader 2, which had a Cohen’s kappa of 0.36 across cohorts.

### Subgroup analysis of radiographers

**Fig. 4:**
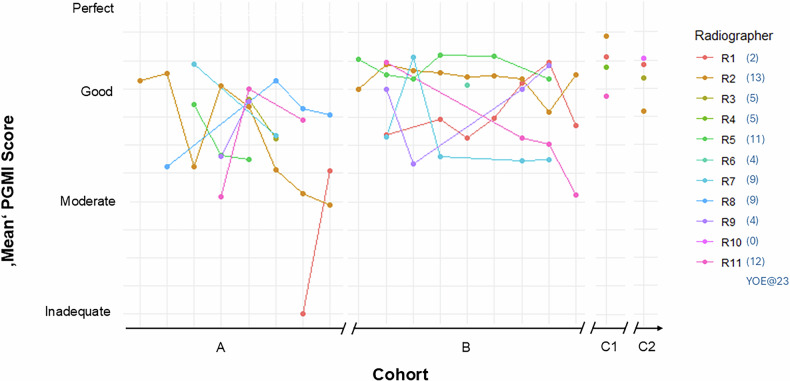
Displays the average PGMI scores of individual radiographers (R1–11) per week on the *y*-axis (1 = ‘Inadequate’; 4 = ‘Perfect’) over time on the *x*-axis, representing different cohorts (A, B, C1, and C2). Each radiographer’s YOE as of 2023 is indicated in brackets

Before the implementation of the PGMI-AI model (Cohort A), radiographers exhibited varying proficiency levels due to differences in education and experience. Following the model’s introduction, notable performance changes were observed, summarised in Fig. 4.

Only two of eleven radiographers participated consistently across all cohorts (A, B, C1, and C2), providing valuable longitudinal data. Radiographer 1 (R1) started in 2021, while Radiographer 2 (R2) had 11 YOE by then. Linear regression analysis for R1 revealed a positive slope of 0.26, indicating a significant performance improvement post-AI implementation. R2 also improved, with a positive slope of 0.18, highlighting the AI model’s positive impact. Residual standard errors remained consistent for both R1 and R2, suggesting that some variability in PGMI scores was not explained by different cohorts alone. Their multiple R-squared values (0.05 for R1 and 0.02 for R2) indicate that the AI model explains a small proportion of the variance in individual quality across cohorts. In contrast, Radiographer 3 (R3), who had a break between cohorts A and C2, showed a lower multiple R-squared value (0.01), indicating minimal variance in scores explained by cohorts.

To assess whether improvements were solely due to increased experience, we compared the mean YOE across cohorts (see Supplementary Fig. S1: Relationship between radiographers’ YOE and PGMI scores for detailed analysis). Cohort A had a mean of 7.83 years (95% CI: 7.67–7.98), Cohort B had 7.86 (95% CI: 7.70–8.03), Cohorts C1 had 6.23 years (95% CI: 5.65–6.81) and Cohort C2 had 5.15 (95% CI: 5.15–6.33). Comparing models with and without YOE, AIC was slightly lower for the model excluding YOE (10,900.34 vs 10,907.82), suggesting that YOE might not significantly improve the model fit. The EMMs of 3.40 for C1, 3.07 for B, 3.06 for C2, and 2.69 for A. Pairwise comparisons revealed significant differences between Cohort A and all other cohorts (e.g. A vs C1: −0.722, *p* < 0.0001). These results suggest that the observed improvements were primarily influenced by the introduction of the AI model, with experience playing a secondary role.

## Discussion

Over a two-year period, the AI application of the b-box^TM^ software demonstrated a stable increase in the frequency of ‘Perfect’ mammograms and a reduction in the number of ‘Inadequate’ mammograms. As AI-driven interpretation becomes more common in breast cancer screening, the need for automated quality assurance grows to ensure consistent imaging standards [13].

Our study supports the notion that AI can contribute to reducing subjectivity in breast positioning assessment, contributing to more reliable image quality [14], particularly for synthetic DBT.

The short-term improvements obtained through AI monitoring were comparable to those obtained through specialised PGMI training by Santer et al, as evidenced by our study’s increase in ‘Perfect’ mammograms from 22.34% to as high as 47.21% [7].

Real-time feedback had a positive effect on radiographer performance, which is in line with a recent study of Eby et al [15]. However, in our current study, a higher percentage of ‘Perfect’ or ‘Moderate’ assessments were observed. In addition, we were able to show a continuous improvement over a two-year period, as required by the FDA’s Mammography Quality Standards Act (MQSA) [15]. These quality improvements [16] may lead to a significant reduction in technical repeats and recalls [17], while also enhancing cancer detection rates [18].

In a survey conducted by Michalopoulou et al with members of the European Society of Breast Imaging (EUSOBI), less than one-third of participants engaged in regular performance testing. However, the vast majority agreed that mandatory testing would be beneficial for improving their skills [19].

Beyond improving quality, our study demonstrated a convergence in interrater agreement (κ) over time, suggesting that training, increased case exposure, and accumulated experience may reduce the subjectivity of human quality assessment and enhance the reliability of PGMI scoring.

In discussing limitations, it’s important to note the risk of inaccurate AI recommendations, particularly for inexperienced readers, which may lead to automation bias [20]. The single-institution dataset limits generalizability to other clinical settings. Cohort comparability may have been influenced by patient composition, as institutional scheduling during the holiday period led to fewer postoperative follow-up patients in Cohorts C1 and C2 than in Cohorts A and B. Although radiographers' experience varied across cohorts, it was not a significant driver of the observed quality differences. Still, as our results and those of Waade et al indicate, AI’s quality assessment performance remains limited, with only slight to moderate agreement for PGMI and its subcategories [14]. For training purposes, Reader 2 was exposed to AI-generated visualisations of the pectoral muscle, PNL, nipple in profile, and inframammary fold to refine PGMI scoring. This exposure may have unintentionally increased interrater agreement, potentially introducing confirmation bias, despite blinding during final assessments. Enhanced positioning quality may also stem from heightened awareness associated with being monitored. Moreover, declining adherence to AI recommendations over time may have contributed to image quality variability.

To summarise, the use of b-boxTM software significantly improved mammography positioning based on PGMI criteria, increasing ‘Perfect’ assessments and reducing ‘Inadequate’ images. While AI shows promise in quality assessment, further training is needed to enhance agreement with human evaluations, underscoring its evolving role in mammography.

## Supplementary information

ELECTRONIC SUPPLEMENTARY MATERIAL

## Abbreviations

95% CI: 95% Confidence interval
AI: Artificial intelligence
AIC: Akaike information criterion
CC: Craniocaudal
DBM: Digital breast mammography
DBT: Digital breast tomosynthesis
DCNN: Deep convolutional neural network
EMM: Estimated marginal means
MLO: Mediolateral oblique
PGMI: Perfect-good-moderate-inadequate
PNL: Posterior nipple line
YOE: Years of experience
