# Supplementary material for: Enhancing breast positioning quality through real-time AI feedback
Source: Eur Radiol. 2025 Jul 15;36(1):55–63. doi: 10.1007/s00330-025-11812-w (PMC12712050; doi:10.1007/s00330-025-11812-w)
Supplement: Supplementary file 1 — ELECTRONIC SUPPLEMENTARY MATERIAL [file 330_2025_11812_MOESM1_ESM.pdf]

Enhancing Breast Positioning Quality Through Real-time AI Feedback
ELECTRONIC SUPPLEMENTARY MATERIAL

Cohort	Postoperative	Screening	Symptomatic	Recall
A	40.04% (n=1154)	25.82% (n=744)	25.95% (n=748)	8.19% (n=236)
B	29.52% (n=976)	29.64% (n=980)	30.97% (n=1024)	9.89% (n=326)
C1	8.19% (n=24)	43.69% (n=128)	36.52% (n=107)	11.60% (n=34)
C2	3.10% (n=8)	43.41% (n=112)	40.31% (n=104)	13.18% (n=34)

Supplementary Table S1: Distribution of consecutively acquired mammograms by examination type (postoperative surveillance, screening, symptomatic, and recall imaging) across all cohorts. Percentages are based on the total number of mammograms per cohort. Observed differences reflect variations in routine institutional scheduling practices.

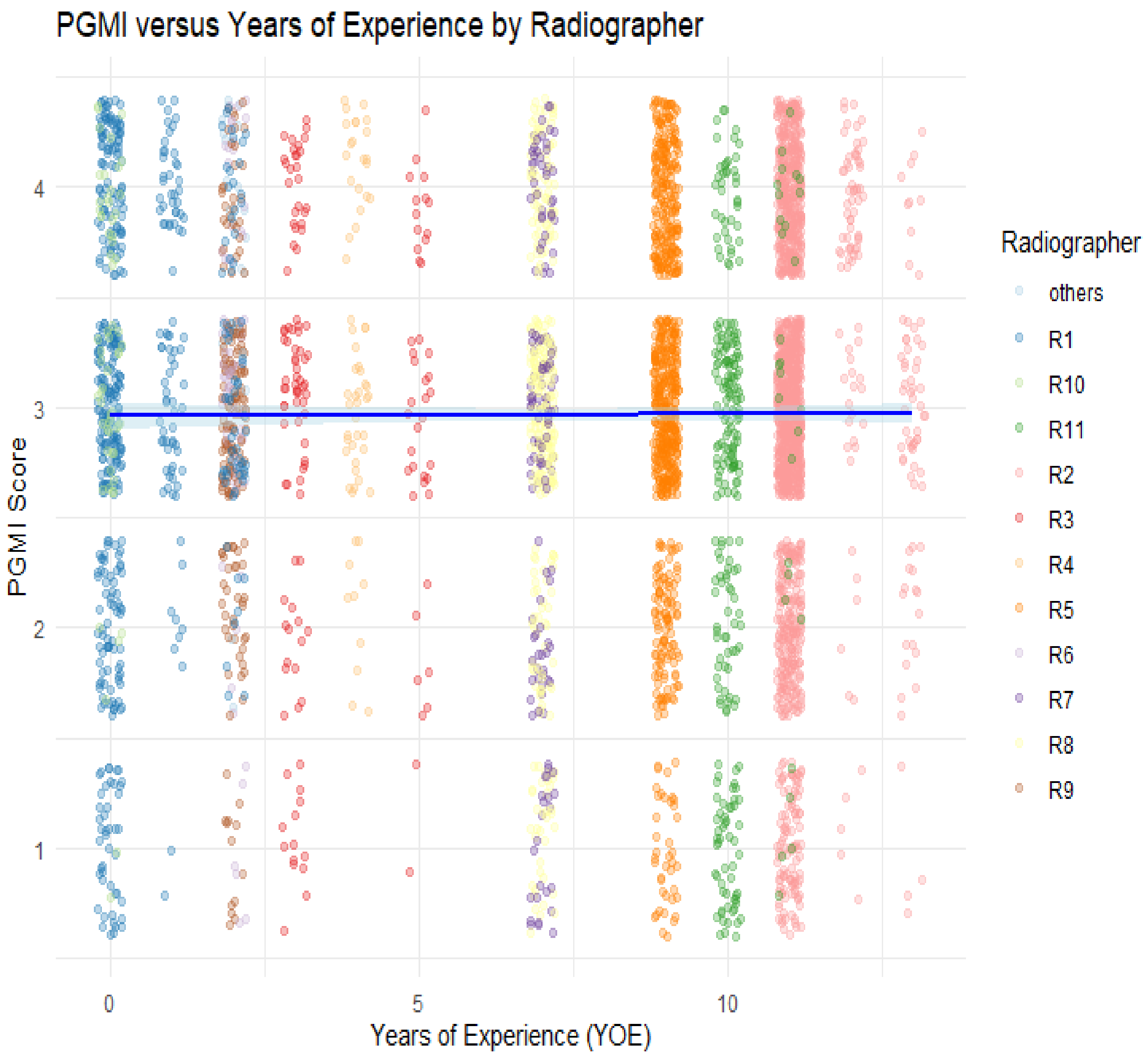

Supplementary Figure S1: Relationship between radiographers' years of experience (YOE) and PGMI scores (Reader 1). Each dot represents an individual mammogram, color-coded by radiographer (R1–R11). A linear regression line is overlaid to show overall trends. Due to the longitudinal nature of the study (2021–2023), YOE increases over time for each radiographer, which explains why some radiographers (e.g., R1) appear at multiple YOE levels (e.g., 0, 1, and 2 years).

Eur Radiol (2025) Sexauer R, Riehle F, Borkowski K, Ruppert C, Potthast S, Schmidt N.
